# Editorial: Sex Differences in Inflammatory Diseases

**DOI:** 10.3389/fphar.2022.962869

**Published:** 2022-07-12

**Authors:** Luigia Trabace, Fiorentina Roviezzo, Antonietta Rossi

**Affiliations:** ^1^ Department of Clinical and Experimental Medicine, University of Foggia, Foggia, Italy; ^2^ Department of Pharmacy, University of Naples Federico II, Naples, Italy

**Keywords:** sex differences, inflammation, autoimmune disorders, cardiovascular system, infectious diseases

Inflammatory diseases significantly differ between men and women in regard to their incidence, manifestations, gravity and prognosis, as well as response to pharmacological treatments (Di Florio et al., 2020; Mauvais-Jarvis et al., 2020). Despite the increasing efforts to consider sex as a crucial biological variable impacting on the pathophysiology of inflammatory and autoimmune diseases, cellular and molecular pathways underlying sexual differences in these disorders remain scarcely elucidated. This is also due to the under-representation of the female sex not only in preclinical research, but also in clinical trials.

In this Research Topic, a team of international experts presents the results of their preclinical and clinical studies focused on sex differences in inflammatory and autoimmune disorders affecting different organs. Moreover, findings regarding sex impact on pathogen-induced immune and autoimmune responses are shown and discussed.

An increasing number of evidence points toward a sex-based effects in several disorders of the cardiovascular system, including autoimmune myocarditis and related cardiac fibrosis and dilated cardiomyopathy (Fairweather et al., 2012; Fairweather et al., 2013). In this regard, in a first original article, Barcena et al. assess possible sex-dependent differences in alterations of cardiac function, inflammation and fibrosis development in an animal model of experimental autoimmune myocarditis, showing the presence of a pro-inflammatory phenotype in male animals and of an anti-inflammatory phenotype in females. Macrophage polarization and activation of cardiac fibroblasts have been reported to be crucially involved in myocardial inflammation and remodeling (Kim et al., 2021). In a second original research article, Barcena et al. demonstrate that the polarization of bone marrow-derived macrophages, with a significant overexpression of M1 and M2 markers, as well as increased levels of reactive oxygen species, specifically occurs in male mice. Interestingly, they also show that an inflammatory environment promotes the activation of cardiac fibroblasts, with significant higher levels of the pro-fibrotic markers TGF-β and IL-1β in activated cardiac male fibroblasts compared to female ones.

Recent data have also highlighted a significant impact of sex-dependent differences in the development of cardiac valve alterations, such as aortic stenosis (AS) (Saeed et al., 2020a; Saeed et al., 2020b). In particular, fibrosis has been shown to mainly affects women’s aortic valves, whereas higher calcification degrees have been described in men. In this regard, Myasoedova et al. evaluate aortic valve fibrosis in men and women with a severe AS, showing a significant effect of sex on the fibro-calcific process of the aortic valve, both at gene expression and cell type level, with lower content of aortic valve calcium, higher fibrosis and an over-representation of mesenchymal cells in women. Pro-inflammatory pathways, characterized by increased levels of monocytes, macrophages, T and B cells, were, instead, enhanced in men. With respect to vessel autoimmune diseases, anti-neutrophil cytoplasmic antibody (ANCA)-associated vasculitis (AAV) affects multiple organs, including kidney, resulting in ANCA glomerulonephritis (GN). In their retrospective study, Tampe et al. aim to evaluate possible sex differences in patients with AAV and biopsy-proven ANCA GN in both laboratory parameters and histopathological scoring of glomerular and tubulointerstitial lesions, as well as AAV extrarenal manifestations. Although sex was not correlated with short-term clinical AAV course, disease severity and ANCA GN classification, females showed a lower tubulointerstitial inflammation and vasculitis of peritubular capillaries compared to males.

Among autoimmune disorders, psoriasis is the one in which the role of sex has been poorly investigated so far, although it has been widely reported that fluctuations of estrogen levels are implicated in the variety of psoriasis manifestation in women (Ceovic et al., 2013). In this regard, by developing an innate immunity dependent mannan-induced psoriasis model, Wu et al. show an increased severity of the disease in female mice of different strains, as well as a prominent expression of estrogen receptor-β on keratinocytes. Moreover, the expression of genes promoting skin inflammation, including the ones for some specific cytokines, i.e. TNF-α, IL-6, IL-22, IL-23, and IL-17 family, was affected by estrogen levels.

Sex impact on the susceptibility to many infectious diseases, as well as on pathogen-induced immune responses and post-infection viral autoimmunity, has gained increasing attention in last years (Ruggieri et al., 2016), especially following the COVID-19 pandemic (Gebhard et al., 2020; Haitao et al., 2020). In this context, a considerable number of factors has been investigated and proposed to be crucially implicated. In their review, Popescu et al. categorize the highly heterogeneous available literature about the involvement of environmental factors, such as endocrine disrupting chemicals, describing and discussing how they can interfere with immune-related endocrine signaling and contribute to autoimmunity and autoreactivity following infections from Epstein-Barr and Herpes Simplex viruses, as well as SARS-CoV-2. In an interesting HYPOTHESIS AND THEORY article, Spiering and de Vries suggest that females may be significantly protected against severe COVID-19, due to the biallelic Toll-Like Receptor 7, one of the crucial recognition receptors for SARS-CoV-2 ssRNA, resulting in a stronger and more protective interferon-mediated response immediately after infection. In their original research article, Kuipery et al. highlight the impact of sex on the responsiveness of liver myeloid cells following Hepatitis B Virus infection, showing more frequent and severe liver damage, together with increased levels of inflammatory markers of myeloid activation, in men, whereas sex did not impact on the frequency or phenotype of sinusoidal myeloid cells. An evaluation of sex effects on lipid mediators (LMs) of inflammation in a rodent model of inflammatory peritonitis, obtained by zymosan administration in male and female mice after gonadectomy, has been conducted in the original research article by Troisi et al. that shows a predominance of specific pro-inflammatory products in the exudates of males, thus revealing sex differences and a clear role of sex hormones in LM biosynthetic networks during acute self-resolving inflammation. Moreover, genetic factors modifying in a sex-dependent way the response to infectious diseases are discussed in a minireview by Lipoldová and Demant, focusing on 22 autosomal genes/loci affecting, in rodent models, the susceptibility to infections from different pathogens only in females or only in males or in both sexes, but with opposite effects.

The body’s response to infections can be severe and result in sepsis that has been described as the leading cause of death in intensive care units, especially when occurring after a trauma (Ma et al., 2016). With respect to this pathological condition, the impact of sex on mortality remains still controversial. In this regard, the retrospective cohort study of Kondo et al., conducted on hospitalized patients with sepsis after trauma, points toward a significant increase of the survival rate in female subjects compared to males, although it is concluded that molecular mechanisms underlying this sex difference need to be further elucidated. On the contrary, the original research article by Scott et al., realized by using a mechanical rat model of traumatic brain injury (TBI), a pathological condition which strongly solicits the immune system (Bao et al., 2021), reports only limited sex-dependent differences in the observed alterations of the blood brain barrier permeability caused by TBI-induced neuroinflammation.

In conclusion, we hope that this Research Topic will be useful for all researchers working in the field of inflammatory and autoimmune diseases and will prompt them to take into adequate consideration possible sex-based differences when designing their studies, in order to obtain reliable results and also to allow a sound translation of preclinical findings into the clinical settings.

We take the occasion to thank the Authors for their outstanding contributions and the Reviewers for their time, comments and suggestions.
